# Catalytic and Structural Characterization of a Browning-Related Protein in Oriental Sweet Melon (*Cucumis Melo* var. *Makuwa* Makino)

**DOI:** 10.3389/fchem.2018.00354

**Published:** 2018-08-23

**Authors:** Siyu Liu, Ayesha Murtaza, Yan Liu, Wanfeng Hu, Xiaoyun Xu, Siyi Pan

**Affiliations:** ^1^College of Food Science and Technology, Huazhong Agricultural University, Wuhan, China; ^2^State Key Laboratory of Chemical Oncogenomics, Key Laboratory of Chemical Genomics, Shenzhen Graduate School, Peking University, Shenzhen, China; ^3^Key Laboratory of Environment Correlative Dietology, Huazhong Agricultural University, Ministry of Education, Wuhan, China; ^4^State Key Laboratory of Bioreactor Engineering, East China University of Science and Technology, Shanghai, China

**Keywords:** browning-related protein (BRP), polyphenol oxidase (PPO), purification, melon, structural characterization

## Abstract

Polyphenol oxidase (PPO) in plants plays an important role in browning reactions and may affect the quality of sweet melon products. In this study, a browning-related protein (BRP) with PPO activity was partially purified from oriental sweet melon (*Cucumis melo* var. *makuwa* Makino) by salt precipitation and column chromatography. The BRP possessed a high degree of identity with several chitinase proteins, particularly defense-related proteins, by MS identification. Pyrogallol was determined as the most appropriate substrate for BRP (*K*_*m*_ = 0.04278 M). BRP exhibited extreme resistance under alkaline and high temperature conditions when pyrogallol was used as substrate. Polyacrylamide gel electrophoresis (PAGE) analysis indicated that BRP was a homo-dimer of two subunits and had a molecular weight of 37 kDa. Structural analysis indicated that the α-helix was the dominant conformation of BRP. The active site of the protein might be buried deeply in the protein, and BRP might be monodispersed in an aqueous system.

## Introduction

Oriental sweet melon (*Cucumis melo* var. *makuwa* Makino) commonly known as sweet melon is widely cultivated in Asian countries. For the favorable growth of this plant, hot and dry climate with large difference in day and night temperature is required (Liu et al., 2017). Sweet melon are considered as highly valued fruits because of high nutritional value and good flavor, thus making this very suitable fruit for juice industry (Fonteles et al., 2012). Meanwhile, a growing consumer demand is observed for high-quality products that retain their fresh-like flavor, overall appearance, and nutritional quality (Liu et al., 2015). During storage and processing operation, enzymatic browning causes serious damage to fruit quality, leading to acceleration of quality deterioration and shelf-life reduction (Chisari et al., 2008). The browning phenomenon in sweet melon products is mostly due to chemical reactions catalyzed by oxidative enzymes.

Polyphenol oxidase enzyme (PPO; EC 1.10.3.1) is widely distributed in plants. It plays a significant role in browning reactions and possesses a di-nuclear copper center (Yoruk and Marshall, 2003). The catalytic copper center is bounded by helical bundles and located near the surface in a hydrophobic pocket (Klabunde et al., 1998). PPO reacts as catalyst to oxidize ortho-diphenols into *o*-quinones by using molecular oxygen and consequently making water due to reduction of oxygen (Mayer, 2006). These quinones are highly reactive electrophilic molecules, which participate in a series of chemical reactions and are further polymerized into brown pigments (Yoruk and Marshall, 2003). In intact cells of plants, PPO and its substrates exist in the chloroplast thylakoid membranes and vacuoles, respectively (Chisari et al., 2008). However, when cutting or bruising occurs during processing, the enzyme and substrates in the cells may come into contact, resulting in rapid oxidation of phenols (Chisari et al., 2008). This reaction deteriorates the product quality by harming color, flavor, and nutritional value. Hence, the catalytic behavior and partial structural characteristics of some browning-related proteins (BRP) are crucial to be determined using biophysical and biochemical techniques to elucidate the catalytic browning mechanism in melons.

Researchers have studied PPO from various cultivars, such as *Satsuma mandarin* (Cheng et al., 2014), banana (Yang et al., 2000), jackfruit (Tao et al., 2013), and persimmon (Navarro et al., 2014). Chisari and others (Chisari et al., 2008) investigated the catalytic characterization of crude PPO and peroxidase (POD) from melons (*C. melo* L.). Rodríguez-López and others (Rodríguez-López et al., 2000) also investigated the purified peroxidase from melon (*C. melo* L.). However, to our knowledge, no data are available regarding the catalytic behavior and structural characterization of purified protein with PPO activity from oriental sweet melon (*C. melo* var. *makuwa* Makino). In the present study, to develop a proper procedure for BRP inactivation, BRP was partially purified from oriental sweet melon samples and was identified through LC–ESI–MS/MS, subsequently, such catalytic properties including substrate specificity, pH stability, temperature stability, and optimum reaction conditions were characterized. The partial structural characteristics were determined using spectroscopy techniques.

## Materials and methods

### Materials and chemicals

Fresh oriental sweet melons (*C. melo* var. *makuwa* Makino) at optimum maturity were purchased from a native market in Wuhan, China. All reagents used were of analytical grade. Ultra-pure water (18 MΩ) from the Milli-Q purification system (Millipore Ibérica, Madrid, Spain) was used to make buffer solutions. DEAE Sepharose Fast Flow column and Sephacryl S200 resin were attained from G.E. Healthcare Amersham (Milan, Italy).

### Protein extraction

All extraction and purification steps were performed at 4°C. Sweet melons (500 g) were homogenized with 0.5 M Tris-HCl buffer (500 mL) containing 50 g L^−1^ of insoluble high-molecular-weight polyvinylpyrrolidone (PVPP) and 0.1% (w/w) ascorbic acid by a juicer (Supor group, Zhejiang province, China). The homogenate was stored at 4°C for 12 h for extraction, then filtered through four layers of cheesecloth and centrifuged at 2,057 × *g* at 4°C for 30 min using a refrigerated centrifuge (5804 R, Eppendorf, Hamburg, Germany). The supernatant was collected as a crude protein solution. Then, the crude protein solution was fractionated by 30% saturation of solid ammonium sulfate to remove impurities, and then 80% saturation was used for precipitation of proteins with PPO activity. The resultant precipitate was resuspended in Tris-HCl buffer (0.5 M, pH 7.0) and dialyzed against same buffer for 24 h, changing the buffer every hour during dialysis. The dialysate was centrifuged at 15,557 × g for 5 min to remove insoluble impurities. The supernatant having the protein was concentrated by a stirred ultrafiltration cup—Amicon® 8050 (Millipore Corp., Bedford, MA, USA).

### Protein purification

The concentrated extract was purified using DEAE Sepharose Fast Flow column (2.6 cm × 25 cm), which was pre-equilibrated with (0.05 M) Tris-HCl buffer (pH 7.0). The protein was eluted with a flow rate of 1 mL per min with a linear gradient of same buffer containing 0–0.5 M sodium chloride at a flow rate of 1 mL/min, collecting fractions per 6.0 mL. The fractions were further collected for PPO activity assay. The highest PPO activity containing fractions were collected, concentrated, and loaded onto a Sephacryl S200 column (1.6 × 90 cm) according to our previous study of Cheng et al. (2014). The PPO activity containing protein was eluted with the same buffer at 0.4 mL per min, collecting for 3.0 mL per fractions. The highest PPO activity containing fractions were pooled, concentrated, and stored in refrigerator at 4°C as BRP sample.

### Measurement of PPO activity

The assay of PPO activity was measured at λ_420nm_(25°C) by increasing the absorbance rate and according to the slope of absorbance value variation was obtained. Two hundred microliter substrate containing 0.05 M pyrogallol in 0.05 M Tris-HCl buffer (pH 7.0) solution, was placed into an enzyme linked immunosorbent assay (ELISA) plate. Afterward, sample solution (50 μL) was added into the plate and quickly analyzed using Multiskan FC (Thermo Scientific, Waltham, MA, USA) at λ = 420 nm with a 10 s interval, reaction time of 90 s. The slope of the curve between the time and concentration of substrate was calculated. The protein sample concentration was determined by “*2.5*. *Protein content*.” PPO activity was assayed in triplicate and converted into relative activity by setting the maximum PPO activity to 100%.

### Protein content

The concentration of protein contents was determined in triplicate based on Bradford method (Bradford, 1976). BSA was used as a standard for this measurement.

### Electrophoresis analysis

To evaluate the protein purity after purification, sodium dodecyl sulfate-polyacrylamide gel electrophoresis (SDS-PAGE) was used using discontinuous electrophoresis method (Schägger and von Jagow, 1991), pH 8.3, in the concentration gel (250 V, 15 mA) and 250 V and 30 mA in the separation gel with a Mini- Protean 4 cell system (Bio-Rad, Hercules, CA, USA). The 2% SDS and β-mercaptoethanol was mixed in the sample and was heated for 5 min in the boiling water. Protein was stained with 0.1 M of (50 mL) Coomassie brilliant blue R-250.

Non denaturing PAGE (Native-PAGE) was conducted using similarly electrophoresis parameters with mentioned above, except for the heating procedure; SDS and β-mercaptoethanol were not added into the sample solution and the gel didn't contain SDS as well (Schägger and von Jagow, 1991). After running, half gel was stained with 5% pyrogallic acid and the other half was stained with 0.1 M Coomassie brilliant blue R-250.

### ESI-QUAD-TOF measurement

To identify the purified protein, the band obtained from SDS-PAGE was excised, the in-gel was digested with trypsin, and analyzed by ESI-QUAD-TOF instrument (Cheng et al., 2014). The detected peptide sequence data were performed automatically by database matching against NCBInr protein database (NCBI) using MASCOT database search engine (Matrix Science; version 2.1.1.0; www.matrixscience.com). The search parameters were set as follows: trypsin digestion with one missed tryptic cleavage site, methionines carbamidomethyl for cysteines, and oxidation for methionines as variable modifications. The sequence peptides with high matching score were aligned by using CLUSTALX software (version 2.0) (Larkin et al., 2007).

### Structural modeling

The peptide sequences obtained from MS were applied for structural modeling. Normal modeling mode of the Protein Homology/analogy Recognition Engine (Phyre) Version 2.0 (http://www.sbg.bio.ic.ac.uk/phyre2/html/page.cgi?id=index) was used for structural modeling (Kelley et al., 2015). The final model and top 10 templates were analyzed and visualized by PyMOL (http://pymol.org/) (Delano, 2002). The crystal structure of sweet potatoes PPO (PDB entry: 1BT1) was selected to compare the active sites with our models (Klabunde et al., 1998).

### Substrate specificity

To determine more affinitive substrate for BRP, Michaelis–Menten constant (K_m_) was determined. Five substrates, such as catechol, resorcinol, pyrogallol, chlorogenic acid, and gallic acid were used at several concentrations of 0.01, 0.02, 0.03, 0.04, and 0.05 M, respectively (Cheng et al., 2014). Fifty millimolar Tris-HCl buffer at pH 7.0 was used to dissolve the substrates. The activity of the sample was determined according to “*2.4. Measurement of PPO activity*.” *K*_*m*_ was calculated based on Mdluli' s method (Mdluli, 2005). The kinetic parameters (*K*_*m*_ and *V*_max_) were determined using the Lineweaver–Burk plot (Mdluli, 2005). The optimal protein substrate could be achieved based on the constant *K*_*m*_.

### Optimal pH and pH stability

To explore the optimum pH for PPO activity, 0.05 M pyrogallol solutions were prepared as substrate solutions using appropriate buffers (0.1 M sodium citrate buffers for pH 3.5–5.5, 0.1 M sodium phosphate buffers for pH 5.0–7.5, and 0.1 M Tris-HCL buffers for pH 7.0–9.0). The catalytic activity was determined according to “*2.4. Measurement of PPO activity*.” Considering the auto-oxidation of pyrogallol (Marklund and Marklund, 1974), the actual PPO activity was the catalytic activity subtracting the rate of auto-oxidation of pyrogallol at the corresponding pH values. To evaluate the pH stability of protein, BRP was incubated in the buffer solutions described above at 4°C for 36 h. Subsequently, the protein solutions were dialyzed to neutral pH against 0.5 M Tris-HCl buffer (pH 7.0) for 4 h. The PPO activity of the protein was determined based on “*2.4. Measurement of PPO activity*.”

### Optimal temperature and temperature stability

The optimum temperature for BRP was measured according to the method described in “*2.4. Measurement of PPO activity*” using spectrophotometry (Eppendorf BioSpectrometer, Eppendorf AG, Hamburg, Germen) at incremental temperatures of 25, 35, 45, 55, 65, 75, 85, and 95°C.

To measure the temperature stability, the sample solutions were incubated at 25, 35, 45, 55, 65, 75, and 85°C in the hot water bath for 15, 25, 35, and 45 min, respectively, and then quickly cooled. The PPO activity of the protein was determined based on “*2.4. Measurement of PPO activity*.”

### Circular dichroism

A concentration of protein solution (0.026 mg/mL) was subjected to circular dichroism (CD) spectral measurements using JASCO J-815 chiroptical spectrometer (Japan Spectroscopic Co., Tokyo, Japan). Spectral units were turned into molar extinction coefficient difference (Δε, M^−1^cm^−1^). The secondary structural contents of proteins were calculated by the Protein Secondary Structure Estimation software of Spectra Manager (JASCO, Japan) based on Yang's reference (Lees et al., 2006).

The parameters of CD were set as follows: the scan speed, time constant, and bandwidth were 120 nm/min, 2 s, and 1 nm, respectively. All tests were carried out within the linear range of the photomultiplier tube voltage. The data were collected three times per line.

### PSD (particle size distribution) measurement

The sample solution was subjected to PSD using Zetasizer Nano-ZS dynamic light scattering device (Malvern Instruments, Malvern, Worcestershire, U.K.). The scattered light at 532 nm wavelength laser, and the reflection angle of laser is 15° (Cheng et al., 2014). The size measurements were exported as the mean of at 10 readings. All analysis was performed in three replicates.

### Fluorescence spectral analysis

Fluorescence spectra were performed according to our former study (Murtaza et al., 2018) by using fluorescence spectrophotometer. Analysis were conducted in the concentration range where emission was linear corresponding to concentrations of fluorophore. The protein sample was measured by fluorescence emission wavelength at 350 nm in the first scan to achieve the (λ_max)_ maximum excitation wavelength, and then the sample was scanned at the maximum excitation wavelength to obtain all emission spectra.

The excitation spectra under the operating conditions were set as follows: maximal excitation wavelength (λ_ex_ = 280 nm) and emission spectra of protein solution (λ_em_ = 300–400 nm); Both emission (Em) and excitation (Ex) slits were set at 5 nm. The speed at 240 nm/min and response time were set at 0.1 s with 1 nm step length for capturing data.

### Statistical analysis

All analysis was carried out in triplicate. The data using “descriptive statistics” and ANOVA (analysis of variance) were analyzed by the Origin 9.2 software. Moreover, *t*-test was applied to determine the differences between the means (*P* ≤ 0.05) and statistical significance.

## Results and discussion

### Protein purification

**Figure 1:**
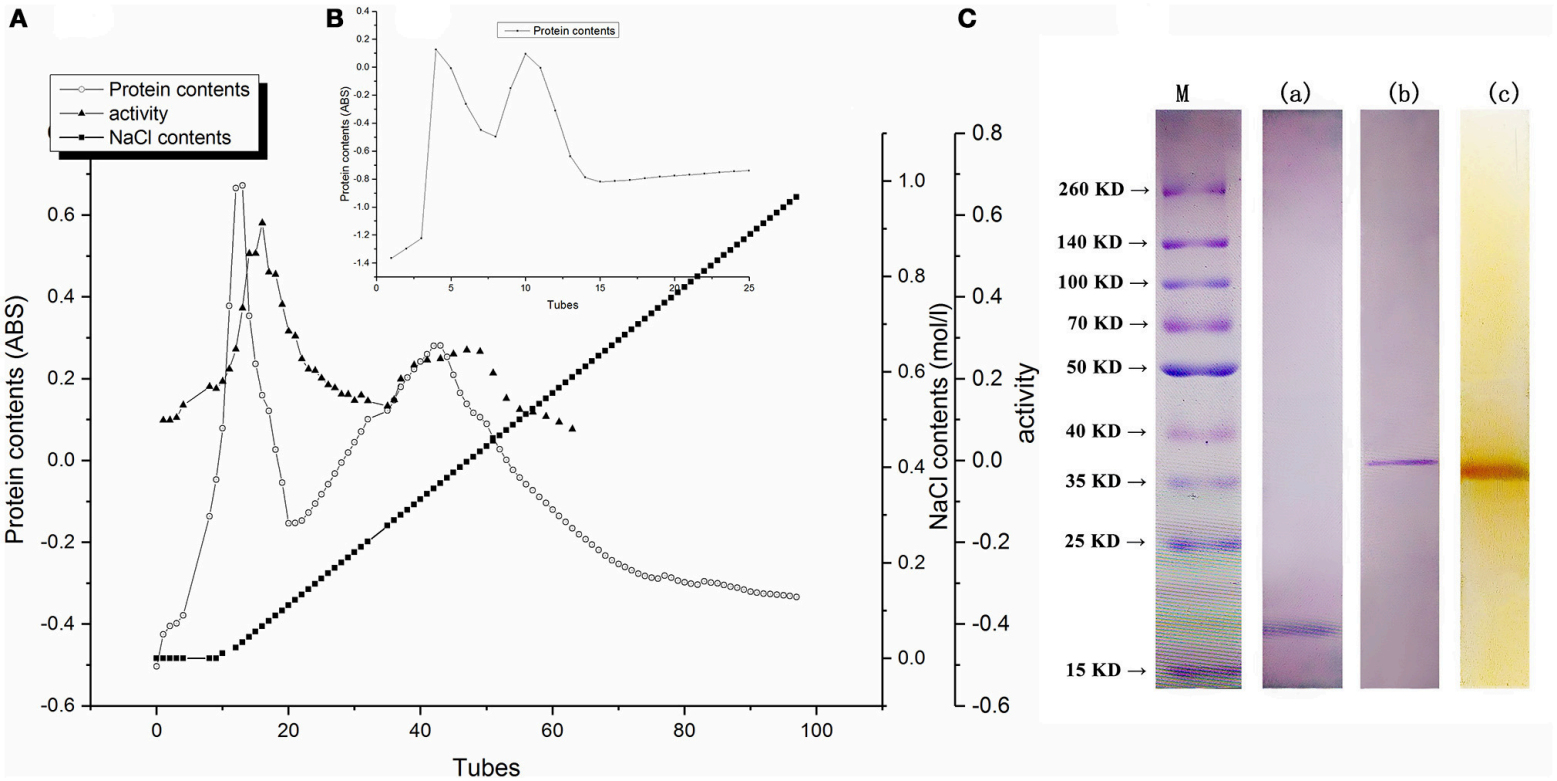
**(A)** Proteins of eluted profiles from DEAE Sepharose fast flow column. **(B)** Profiles of BRP eluted from Sephacryl S200 column. **(C)** Electrophoresis behavior of BRP. Lane a, SDS-PAGE of BRP stained by Coomassie Brilliant Blue R-250. Lane b, Native-PAGE of BRP stained by Coomassie Brilliant Blue R-250. Lane c, Native-PAGE of BRP stained by pyrogallol. Lane M, Prestained protein ladder.

As shown in Figure 1A, fractions peak with higher activity of PPO were eluted with constantly increasing salt concentration. Two peaks with higher PPO activity were detected. The fractions containing highest protein concentration are consistent with the peak fractions with highest activity of PPO. However, the range of the second peak was extremely broad that the protein might be impure. Meanwhile, the PPO activity of the second peak fraction was much lower than the first one, indicating that the first protein peak might be decisive in enzymatic browning. These fractions of the first peak were subjected to further purification by using Sephacryl S200 column; the elution profile is shown in Figure 1B. The maximum protein amount and highest PPO activity were detected in the second peak (data not shown), and these were eluted and collected for PAGE analysis. As shown in Figure 1C, -PAGE and Native-PAGE upon staining with Coomassie brilliant blue revealed single band from purified protein, indicating homogeneity of the PPO protein. Furthermore, activity assay was also performed by using substrate pyrogallol, a clear brown band was observed at the same position by staining with Coomassie brilliant blue R-250, indicating the strong PPO activity of BRP and confirming that the purified protein was the desired one with highest PPO activity.

As shown in Figure 1C, the molecular weight of purified BRP was approximately 18 kDa determined through SDS-PAGE analysis and 37 kDa detected through native PAGE analysis. These results indicated that the protein was a homo-dimer of two subunits. The molecular weights and existing structural forms of PPOs showed a wide variation depending on the enzyme source (Yoruk and Marshall, 2003). For example, PPO was depicted as a 108-kDa pentamer in *Satsuma mandarin* (Cheng et al., 2014), a 42-kDa monomer in banana (Yang et al., 2000) and as a 130-kDa dimer from jackfruit (Tao et al., 2013). It was hypothesized that enzyme active center of PPO protein might be present in cavity surrounding by subunits or may be positioned in hydrophobic region to form active center of PPO activity (Cheng et al., 2014).

### LC–ESI–MS/MS identification and structural modeling analysis

**Figure 2:**
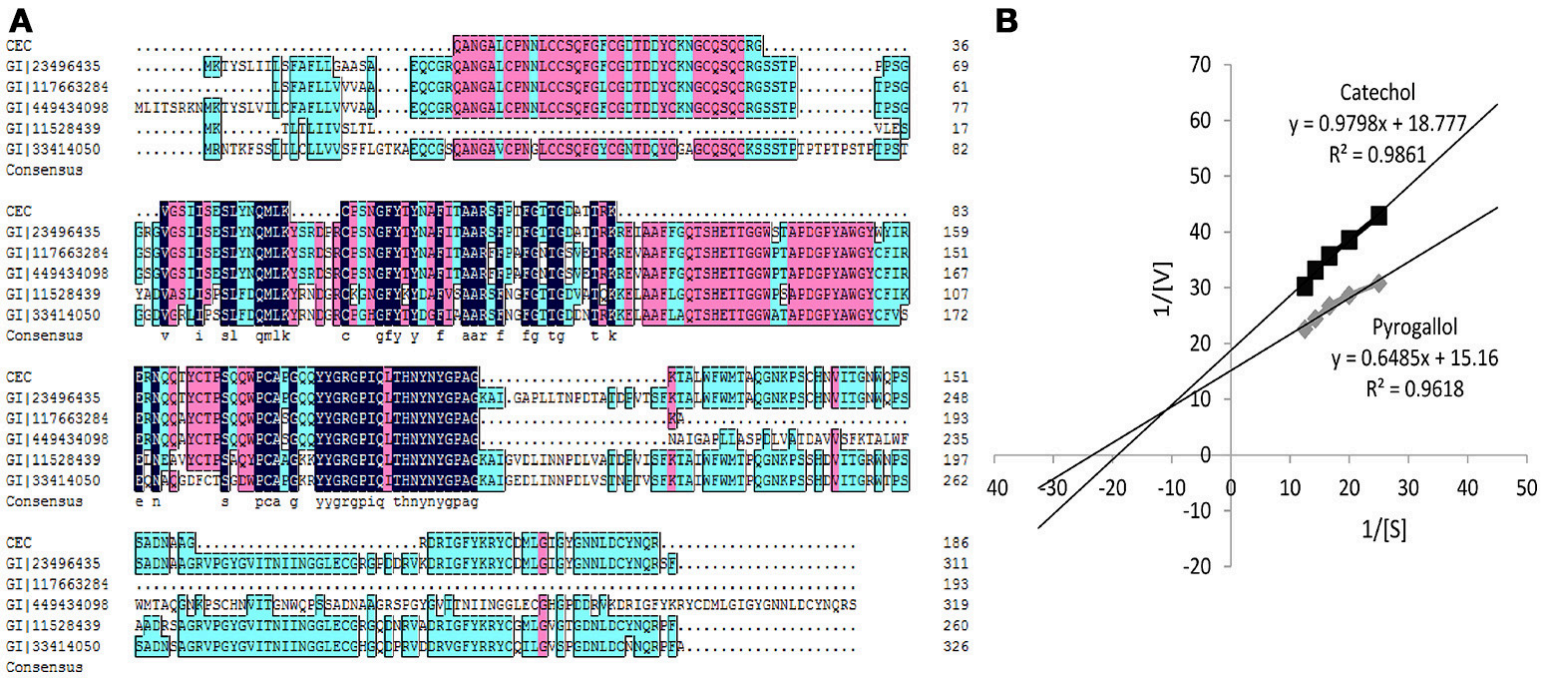
**(A)** Peptides sequenced alignment of BRP purified from sweet melons. **(B)** *Km* values of catechol and pyrogallol substrates for BRP. The vertically intercept was 1/*V*_*max*_ while the horizontally intercept was 1/*K*_*m*_. All data were the means of three independent experiments (*P* < 0.05, *n* = 3).

As shown in Figure 2A, some peptides of BRP in sweet melon (*C. melo* var. *makuwa* Makino) (marked as CEC) matched with the peptides of endochitinase MCHT-2 in *C. melo* (gi|23496435) and *Cucumis sativus* (gi|117663284). Some conserved sequences exist among five proteins, all of which are referred to as chitinase, such as the YYGRGPIQLTHNYNYGPAG sequence. From the matching perspective, the protein (gi|23496435) holds a higher degree of identity. The matching protein of BRP shares 59% sequence coverage with the protein (gi|23496435) with the matching score of 2,455 points (*p*<*0.05*). Meanwhile, the protein sequence (gi|23496435) provided a calculated nominal mass of 34.8 kDa, which is close to the experimental values (37 kDa) obtained from the purified BRP.

**Figure 3:**
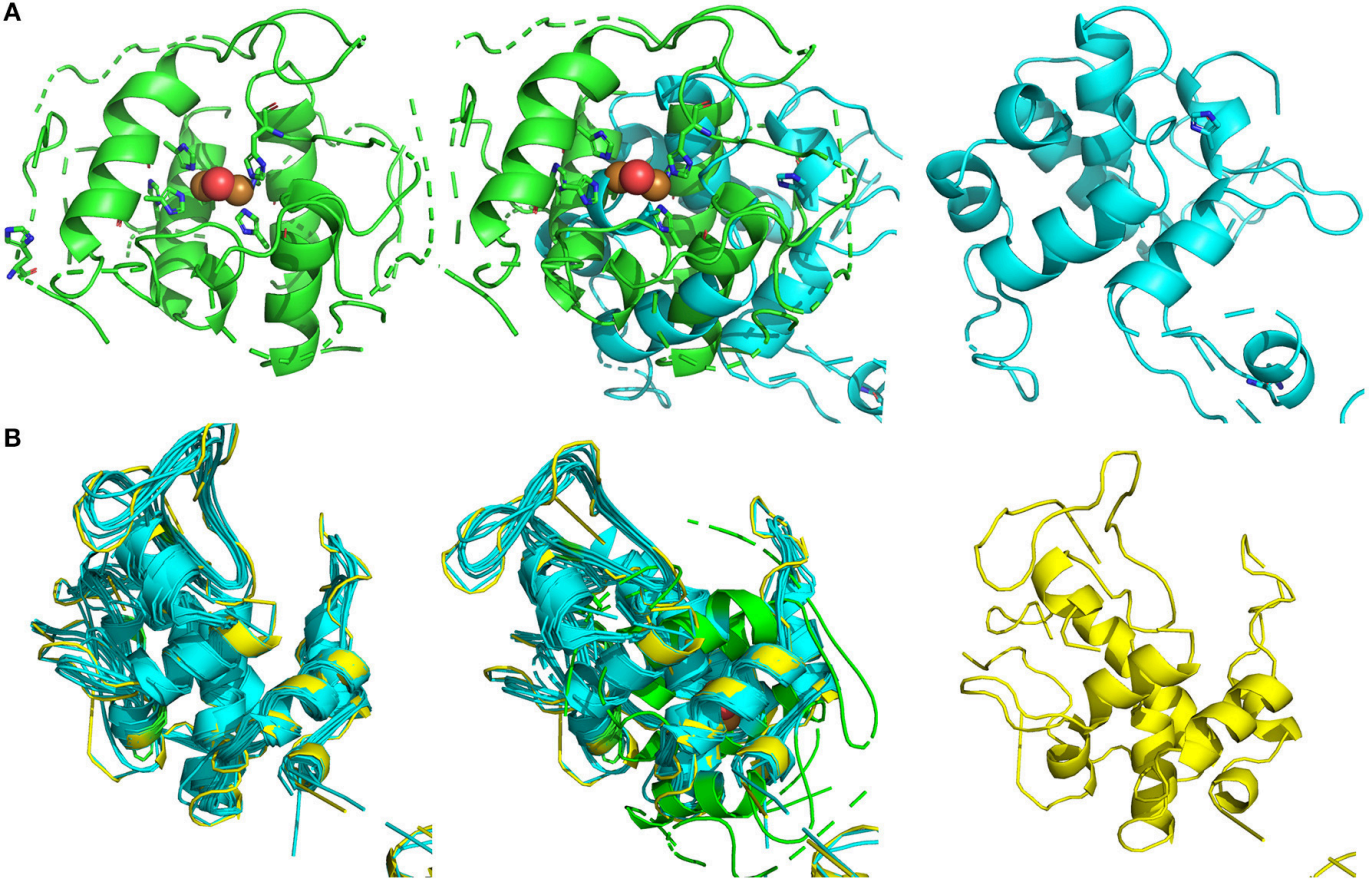
Structural modeling and visualization of three dimension structures. **(A)** Green, crystal structure of sweet potatoes PPO (PDB entry: 1BT1); cyan, final predicted structure of BRP; mixture, model fitting. **(B)** Green, crystal structure of sweet potatoes PPO (PDB entry: 1BT1); cyan, modeling templates (top 10); yellow, final predicted structure of BRP.

By the Phyre2 platform, the sequence obtained from MS was used to construct a homology three-dimensional model. The final structural model of BRP was obtained using 20 best modeled templates, most of which are associated with antimicrobial functions. The top 10 templates which belong to chitinase family possessed more structure information and had a higher similarity (nearly to 100% confidence). The firstly ranking template was the crystal structure of class I chitinase (PDB entry: 2DKV) (Kezuka et al., 2010), which RMS, confidence and identity were 0.001, 100, and 63%, respectively. As shown in Figure 3B, the top 10 templates (colored in cyan) and final model (colored in yellow) were fitted by PyMOL, and these models showed conserved three-dimensional structure with each other (RMS < 1.000). All of them had abundant α-helix bundles.

Endochitinase is a defense protein in plants, which may belong to pathogenesis-related proteins that play vital roles in protecting plants from attack by pathogens and in adapting to adverse environments (Legrand et al., 1987; Bill et al., 2016). Pathogenesis-related protein family is associated with diverse defense functions, such as antifungal activity, β-1, 3-glucanase, chitinase activity, and the polyphenol oxidase activity correlated to this study (Hu, 2011; Cheng et al., 2014). Pathogens or other plant defense elicitors could affect the expression level of these proteins (Bill et al., 2016). Chitinase catalyzes the cleavage of bond between C1 and C4 of two N-acetyl-D-glucosamine monomers of chitin, which is widely found in fungal and bacterial cell walls and exoskeleton of the arthropods (Bill et al., 2016). Therefore, chitinases could collapse the exterior protective structure of pathogens and arthropods and could play a role in the plant defense system.

Similar to chitinase, PPO is involved in a plant defense mechanism against pests and pathogens (Mayer, 2006). When plants are exposed to pests, both PPO genes and chitinase genes could be overexpressed (Mayer, 2006; Bill et al., 2016). The amino acid sequence shows considerable variability among species, and multiple forms and functions of PPO exist in most plants and fungi (Mayer, 2006). PPO was reported to alter the carbohydrate-binding sites of microbial proteins and consequently functions as an anticaries agent (Cowan et al., 2000). Meanwhile, some other enzymes also exist with existing PPO activity. For instance, aureusidin synthase, which is a PPO homolog, exhibited an affinity for the traditional PPO substrate (Nakayama et al., 2001). Rahman and others also introduced that Japanese radish PPO, soybean PPO, and a purified enzyme from cauliflower not only possess PPO activity but also peroxidase activity (Rahman et al., 2012).

From the fitting between final modeled structure of BRP and sweet potato PPO structure (Figure 3A, middle), the predicted BRP showed a similar structure (RMS = 5.069) with the catalytic site of sweet potato PPO, which was constructed by several α-helix bundles (Klabunde et al., 1998). The modeled templates and sweet potato PPO were also conducted to fitting by PyMOL (Figure 3B, middle). The RMS of the fitted models was 11.150. However, the overall structure (Figure 3B, left) of all overlapped templates was also similar with PPO active site, which was abundant of a-helix bundles and formed a cavity for catalyzing the phenol substrate. Thus from the point of structure modeling, the BRP in this research has potential in forming similar catalytic cavity, which contributing to PPO activity.

Similar findings of pathogenesis-related proteins with PPO activity have been found in apple (*Malus domestica*) (Hu, 2011), *Satsuma mandarine* (Cheng et al., 2014), mango, and Jing orange (unpublished works) in our previous study. Our findings implied that a strong relationship exists between chitinase and PPO activity. However, further research must be conducted to explore the possible mechanism behind this phenomenon. To further understand the basis of the functional protein, relevant catalytic and partial structural characteristics were subsequently analyzed.

### Substrate specificity

As shown in Figure 2B and Table 1, the Michaelis constant (*K*_*m*_) and maximum reaction rate (*V*_max_) for BRP of oriental sweet melons with two substrates at various concentrations were calculated by Lineweaver–Burk plot. *K*_*m*_ represents the affinity of the substrates binding to enzymes. The decrease in K_m_ reflects the increase in the affinity for binding (Ünal and Sener, 2016). The *K*_*m*_ values of BRP with catechol and pyrogallol substrates were 0.0522 and 0.0428 M, respectively. Resorcinol, gallic acid, and chlorogenic acid were also considered. However, *K*_*m*_ values estimated for these substrates were in the millimolar range (data not shown), suggesting only weak substrate binding. BRP had higher affinity toward pyrogallol as evidenced by lower *K*_*m*_ value. *V*_max_ value showed that the reaction rates for BRP were higher using pyrogallol, indicating that BRP was more active with pyrogallol. PPO shows higher activity to the substrate with increasing numbers of phenolic hydroxyl (Rice-Evans et al., 1996). Besides, phenolic hydroxyl of ortho position shows higher activities than those of para positions or meta positions (Rice-Evans et al., 1996). Therefore, pyrogallol was the most appropriate substrate for BRP. The results obtained in this study were in agreement with that reported in satsuma mandarine (Cheng et al., 2014). Substrate specificity varies depending on the enzyme (Mayer, 2006). Such substrate specificity of BRP differs from dopamine of PPO in banana (Yang et al., 2000), catechol of PPO in jackfruit (Tao et al., 2013), 4-methyl catechol of membrane-bound PPO in apple (Liu et al., 2015) and o-diphenols of PPO in red globe grape (García-García et al., 2013), which also vary from each other's.

**Table 1:** Determination of K_m_ and V_max_ for BRP.

**Substrate**	**K_m_(mol/l)**	**V_max_(U/min)**	**Slope**	***R*^2^**
Catechol	0.05218	0.05326	0.9798	0.9861
Pyrogallol	0.04278	0.06596	0.6485	0.9618

*All data were the means of three independent experiments (P < 0.05, n = 3)*.

### pH stability and optimal pH

**Figure 4:**
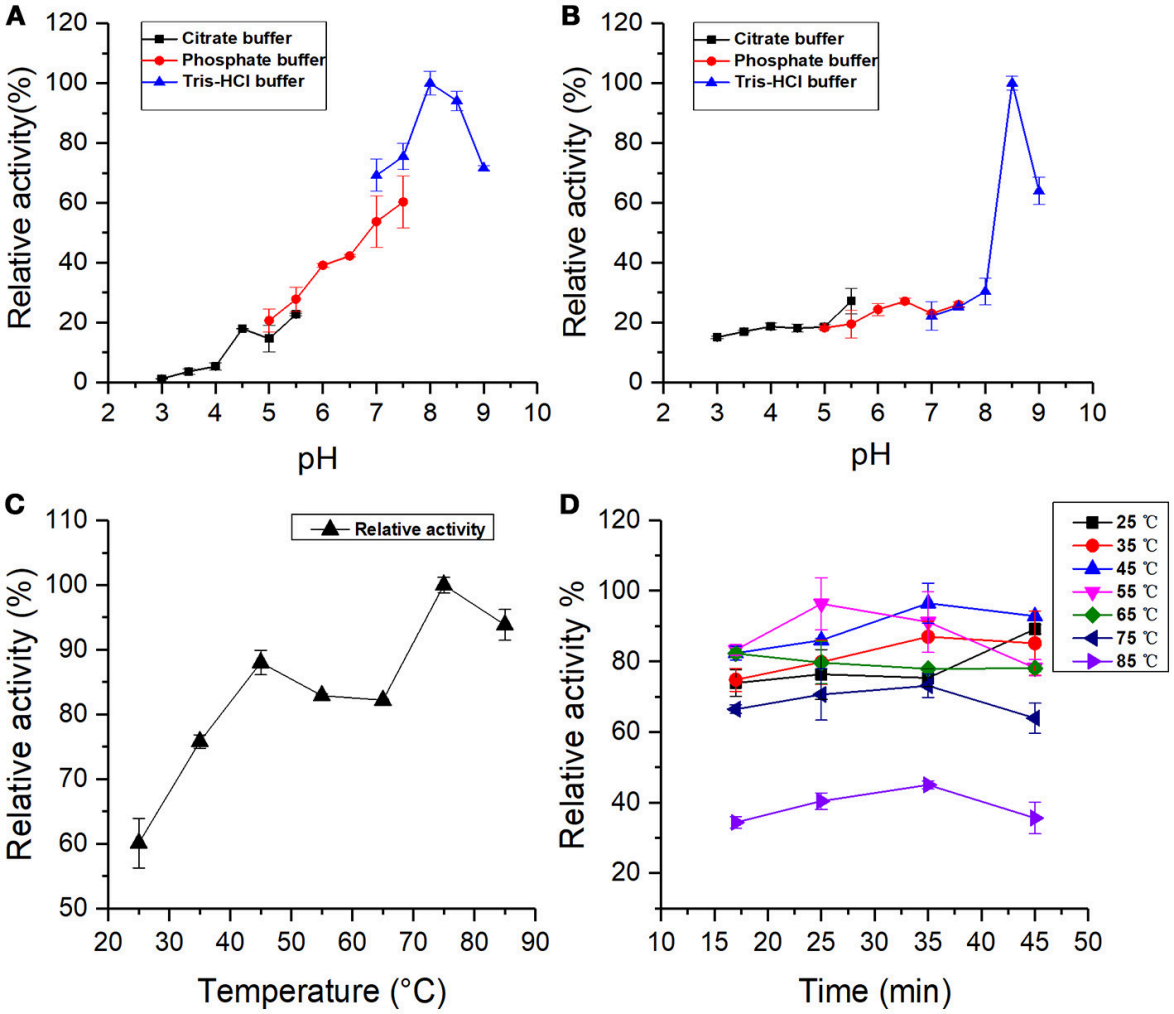
**(A)** pH stability: BRP activity incubated at different pH values for 36 h. **(B)** Optimal pH: BRP activity as a function of pH value. **(C)** Optimal temperature: BRP activity as a function of different temperatures from 25 to 85°C. **(D)** Temperature stability: BRP activity incubated at different temperatures. All data were the means ± SD of three independent experiments (*P* < 0.05, *n* = 3).

The buffer solution of various pH values can change the ionization state of both substrate and amino acid chain, which will affect the combination of enzyme and substrate (Yoruk and Marshall, 2003). The effect of pH on BRP was determined by measuring the PPO activity in buffer solution with pH values from 2.0 to 9.0, using pyrogallol as substrate (Figures 4A,B). Figure 4A shows the pH-stability profile for BRP. In contrast to most PPOs, which are more stable at a neutral pH region (Ng and Wong, 2014), the sweet melon BRP was considered more resistant in alkaline environment. The sweet melon BRP was most stable at pH 8.0, which was an alkaline pH value. Above and below this pH value, the protein was less stable. Even the weak alkaline factors might partly activate the BRP. Chang-Peng Yang and others (Yang et al., 2000) obtained a similar result that PPO from banana (*Musa sapientum* L.) pulp retained more than 90% of the original activity between pH 5 and 11 after 48-h incubation. Liu et al. (2015) also reported that a membrane-bound apple PPO was stable from pH 5.0 to 8.5. Moreover, Rahman et al. (2012) showed that the cauliflower PPO was stable in the pH range of 3.0–11.0. The isoelectric points of most PPOs were in the acidic region, where PPOs would obtain precipitate and result in loss of the activity (Cheng et al., 2014).

The optimal pH profile for the oxidation of pyrogallol by BRP is shown in Figure 4B. The pH-relative activity of BRP exhibited a small shoulder around pH 6.5 followed by a sharp curve at pH 8.5, indicating that BRP had two optimal reaction pH values, one was at pH 6.5 and the other would be activated at pH 8.5. The optimum pH for PPO activity varied with the enzyme source and experimental factors, such as the species of phenolic substrates, extraction methods, and buffer system used during determination (Ünal and Sener, 2016). Liu et al. (2015) also reported that mPPO from Fuji apples had two optimal pH values at 4.0 and pH 8.0. Chisari et al. (2008) and Jose Neptuno Rodríguez-López et al. (2000) found that the optimal pH values for PPO from melon (*C. melo* L) were 7.0 and pH 5.5, which were closer to pH 6.5 in this study.

Optimum alkaline pH values for PPO catalysis were widely reported. Such pH values are 8.0 in Fuji apples (Liu et al., 2015), pH 8.0 in kiwifruit and spinach (Yoruk and Marshall, 2003), and pH 8.0 in cauliflower (Rahman et al., 2012), which were close to the other optimal pH (pH 8.5) in this study. The PPO activity of BRP in alkaline region was much greater than in the acidic one. Marklund and Marklund (Marklund and Marklund, 1974) reported that pyrogallol can auto-oxidate in weakly alkaline solutions to form free radicals and intermediate products, which are highly active in the oxidation process. Considering the auto-oxidation of pyrogallol into various degrees of oxidized intermediates, BRP could more easily catalyze the intermediates in the alkaline region to the subsequent formation of brown pigments. The free radicals induced by the auto-oxidation of pyrogallol can also considerably accelerate the melanin formation (Yoruk and Marshall, 2003). Meanwhile, pH stability assay indicated that BRP remained active and was even activated in the alkaline environment. From the effect of factors described above, the sweet melon BRP exhibited higher activity in alkaline condition than in acidic region. Therefore, acidic conditions can be used as a potential measure to control browning reaction in oriental sweet melons, which is similar to the PPO in persimmon (Navarro et al., 2014).

### Optimal temperature and temperature stability

The profile of variation in the PPO activity with temperature is shown in Figure 4C. Results exhibited that the relative activity of BRP had two peaks at 45 and 75°C. The PPO activity at 75°C was 1.2 times higher than that under 45°C. Liu et al. (2015) also found that both mPPO and sPPO (soluble polyphenol oxidase) from Fuji apples possess two optimal temperature values. The purified PPO in round brinjal was thermal resistant and showed a sudden increase of enzyme activity at 60°C using 4-methylcatechol as substrate after the optimal temperature (30°C) (Ng and Wong, 2014). The optimum temperature for PPO activity exhibits significant variability depending on the substrate and the enzyme source, which were used in the assay (Ünal and Sener, 2016). In this study, the optimal temperature (45°C) of sweet melon BRP was within the range of 30 to 55°C reported in various sources (Yang et al., 2000; Tao et al., 2013; Navarro et al., 2014). The other optimal temperature of 75°C indicated high thermal stability for sweet melon BRP, which is also found in PPOs from banana (Yang et al., 2000), kiwi fruit (Park and Luh, 1985), and head lettuce (Fujita et al., 1991).

Koval et al. (2006) provided synthetic models of the active site of catechol oxidase, which may have several binding sites with copper atoms as catalytic sites. PPO activity may be influenced under ambient conditions by the ability of the substrate binding to the active site of enzymes (García-García et al., 2013). PPO may exist in a latent form, which can be activated through conformational change by treatments, such as exposure to detergents (SDS), acid pH, unsaturated fatty acid, proteases, and heat (Terefe et al., 2010). Proper heating may provide energy and subsequently induce exposure of the PPO active site (Yemenicioglu et al., 1997). From the structural modeling (Figure 3), it' indicated that the structure of BRP might be highly flexible and that rich α-helix bundles could tend to form multi cavities. In the present study, BRP with PPO activity might exist in a latent form, and heat might produce a large amount of energy to form an activated catalytic center, which is also a more favorable conformation for binding with pyrogallol. Meanwhile, in hot environment, the autoxidation of pyrogallol also forms free radical and several intermediate products (Marklund and Marklund, 1974), inducing more intense enzymatic oxidation by BRP. PPOs from round brinjal (Ng and Wong, 2014), carrots (Söderhäll, 1995), and strawberry (Terefe et al., 2010) were also thermal resistant and exist in a latent PPO form, which could cause activation by heating.

The thermal stability of BRP is shown in Figure 4D. The PPO residual activity increased with increasing incubation time at 25, 35, and 45°C and decreased with increasing incubation time at 55 and 65°C. The PPO activity of BRP incubated at 75 and 85°C reached the maximum at 35 min. The activity was reduced by 68% at 85°C for 45 min. In general, BRP incubated at varying temperatures exhibited enzyme activation in the range of 35–55°C and subsequently inactivated with treatment temperatures above 55°C. Proper thermal treatment may import energy for tertiary structure unfolding of the BRP and exposing the active center of the protein, which induced a favorable conformation for binding with substrate. With the heat treatment progressing deeper for longer time, the protein might be denatured and the active center was partially disrupted resulting in the reduction of the relative activity.

### Structural spectrum analysis of the BRP

The variance in hydrophobic areas and aggregation states of proteins, as well as the environmental conditions, all resulted in a certain degree of performances in catalytic properties (Mayer, 2006). The partial structural characteristics of the sweet melon BRP were investigated using CD, fluorescence spectral measurement, and dynamic light scattering (DLS) analysis.

**Figure 5:**
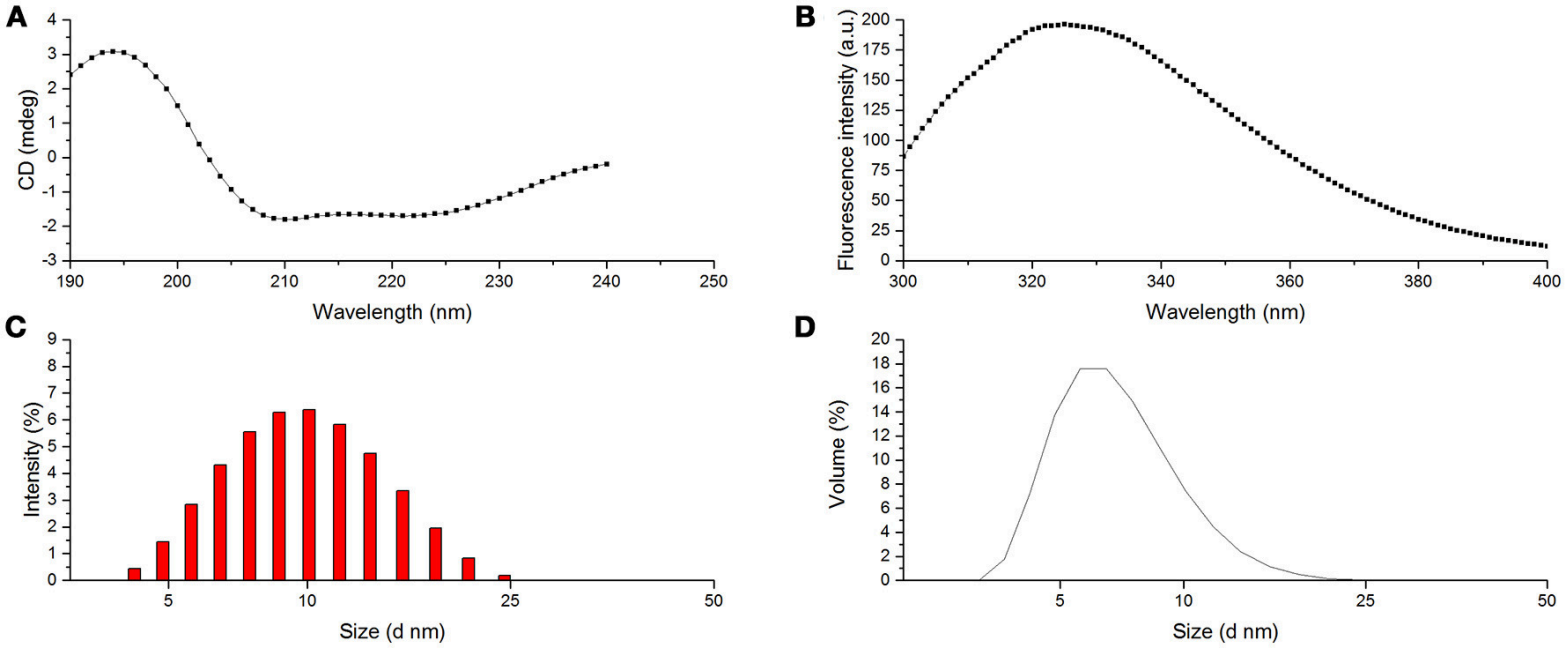
Structural characteristics of BRP. **(A)** CD spectra of BRP; **(B)** Fluorescence spectra of BRP; **(C)** PSD of BRP (intensity); **(D)** PSD of BRP (volume).

Furthermore, the secondary structural composition of proteins was investigated in many cases using far ultraviolet CD spectrum. The typical CD spectrum of α-helix conformation shows a double negative peak at 222 and 208 nm and a positive peak near 190 nm. The β-sheet conformation has a negative peak at 217–218 nm and a strong positive peak at 195–198 nm. Random coil also has a negative peak at 198 nm with a small wide positive peak at 220 nm. The CD spectrum of protein is an integration of these conformations (Lees et al., 2006). As shown in Figure 5A, CD spectra of BRP showed a positive peak at 194 nm and two double negative peaks at 222 and 208 nm, indicating that BRP had a typical conformation of α-helix in the secondary structure. As presented in Table 2, α-helix, β-sheet, β-turn, and random coil contents of BRP were calculated as 53.3, 0, 25.3, and 21.2%. In this study, α-helix was the dominant conformation of BRP, consistent with our structural modeling and the previous study on structural conformation of PPOs (Mayer, 2006). The α-helix is the fundamental conformation in PPOs, which could form bundles surrounding the catalytic copper center (Klabunde et al., 1998). Therefore, the high α-helix structural content of BRP accommodated the catalytic center to be responsible for the performance of PPO activity.

**Table 2:** Secondary structure contents (%) of BRP.

**Protein**	**α-Helix**	**β-Sheet**	**β-Turn**	**Random coil**
BRP	53.5	0	25.3	21.2

The intrinsic fluorescence of protein mainly comes from the residual Trp, Tyr, and Phe (Khan et al., 2013). Among all these residuals, the molar extinction coefficient of Trp is the highest and could be the energy transferring receptor (Khan et al., 2013). From the maximum emission wavelength (λ_*max*_), the environmental information surrounding the Trp residual could be obtained (Khan et al., 2013). As shown in Figure 5B, BRP showed the largest emission peak at 325 nm (λ_*max*_); this result indicated that the fluorophore of this protein was located in hydrophobic environment. Hence, the PPO active site of BRP might be buried deeply in the protein and demand more energy to be exposed outside the surface for the interaction with the substrates, inducing a strong resistance against alkaline or thermal conditions.

DLS is used to measure hydrodynamic sizes, polydispersity, and aggregation effects of protein sample (Park et al., 2004). Figure 5C,D show the particle size distribution (PSD) patterns of BRP. The highest differential intensity for BRP was 6.4% at 10 nm with a relatively narrow span of 4.2–24.4 nm. Figure 5D demonstrates a steep peak value of 17.6% in volume fraction with the corresponding particle diameter of 6.5 nm. The fluorescence spectra also indicated that the hydrophobic residues were buried in the protein. Thus the surface of BRP might be more hydrophilic that BRP in the aqueous system might be monodisperse to perform the catalytic oxidation (Li et al., 2014).

## Conclusion

In this study, an oriental sweet melon BRP with PPO activity was successfully purified through precipitation of ammonium sulfates, ion-exchange chromatography, and gel filtration. BRP was identified by LC–ESI–MS/MS to share a higher degree of identity with endochitinase MCHT-2, which may belong to plant defense proteins. BRP exhibited a higher affinity toward pyrogallol and remained stable above pH 8.0 and temperatures over 75°C. Plant defense proteins should mostly survive harsh conditions during growing and harvesting, even in food preparation and digestion (Smole et al., 2008), which may contribute to the stability of protein to withstand extreme pH environment and thermal treatment. BRP with PPO activity might exist a latent form, which could be activated by more heat or alkaline condition. Based on structural analysis, the active site of protein might be surrounded by high α-helix subunits and buried deeply in the hydrophobic environment, for which more energy was demanded to activate the BRP.

The PPO activity was inhibited in the acidic environment. Thus, acidic treatment can be implemented during the processing of sweet melon fruits to prevent browning. However, a more proper procedure should be proposed to meet the growing consumer demand and acquire wider applications in the inhibition of PPOs. Hence, further research is necessary to determine the influence of several treatments on the inactivation of PPOs and propose more effective treatments applicable on the industrial scale. Meanwhile, more attention could be offered to the mechanism of defense-related protein with PPO activity.

## Author contributions

SL mainly finished the whole data collection and drafted the manuscript. AM helped in writing and analyzing the results. YL assisted in collecting the whole data and statistical analysis. WH and XX designed the study. WH revised the draft and is responsible for corresponding with Journal. SP helped analyzing the data and providing with research supports.

### Conflict of interest statement

The authors declare that the research was conducted in the absence of any commercial or financial relationships that could be construed as a potential conflict of interest.

## Abbreviations

PPO: polyphenol oxidase
BRP: browning-related protein
Tris: tris (hydroxymethyl) aminomethane
PVPP: polyvinylpyrrolidone
PAGE: Polyacrylamide gel electrophoresis
SDS, sodium dodecyl sulfate: sodium salt
DSL: dynamic light scattering
PSD: particle size distribution
CD: circular dichroism.
